# Reliability of Home Sleep Apnea Testing for Diagnosing Obstructive Sleep Apnea in Patients With Spontaneous Cerebrospinal Fluid Leaks

**DOI:** 10.7759/cureus.29854

**Published:** 2022-10-03

**Authors:** James D Johns, Armin Mortazavi, Alexandra Welschmeyer, H. Jeffrey Kim, Suzette K Mikula, Michael Hoa

**Affiliations:** 1 Department of Otolaryngology, Head and Neck Surgery, MedStar Georgetown University Hospital, Washington, D.C., USA; 2 Department of Otolaryngology, Head and Neck Surgery, Georgetown University School of Medicine, Washington, D.C., USA

**Keywords:** spontaneous cerebrospinal fluid leak, polysomnography, obstructive sleep apnea, obesity, home sleep apnea testing

## Abstract

Study Objectives: To establish the prevalence of obstructive sleep apnea (OSA) in patients with spontaneous cerebrospinal fluid (sCSF) leaks and demonstrate the reliability of home sleep apnea testing (HSAT) to screen for OSA in this population.

Methods: A literature review was performed to assess data on OSA prevalence in sCSF leaks. An institutional retrospective review was performed of 20 patients with sCSF leaks who met inclusion criteria. Patients without prior sleep studies were prospectively administered sleep studies, either HSAT or polysomnogram (PSG).

Results: Twenty patients met the inclusion criteria. Two patients had prior sleep studies while 18 patients obtained prospective sleep studies following diagnosis and prior to management of sCSF leaks. Nineteen patients (95%) had evidence of mild or greater OSA.

Conclusions: This study re-demonstrates the high prevalence of OSA in patients with sCSF leaks, consistent with current literature, and investigates the reliability of HSAT for diagnosis of OSA in this population.

## Introduction

Obstructive sleep apnea (OSA) is a disorder of upper airway collapse that results in a number of complications including excessive sleepiness, reduced quality of life, cognitive and functional decline, cardiovascular morbidity, perioperative complications, and death. Although OSA has been increasingly recognized as with associated poor health outcomes, it is estimated that over 85% of patients with clinically significant OSA are undiagnosed [1]. Furthermore, OSA has been implicated in the elevation of intracranial pressure (ICP) which has independently been associated with higher rates of spontaneous CSF (sCSF) leaks [2].

Recent studies have analyzed the relationship between obesity, OSA, and sCSF leaks. A study by Schlosser et al. has reported that a combined 15 of 16 (94%) patients with sCSF leaks were clinically obese [3]. Rabbani et al. performed a prospective polysomnogram (PSG) of patients with sCSF leaks and found 83.3% of patients to have OSA [4]. Furthermore, one study shows an increased prevalence of OSA in sCSF compared to non-spontaneous CSF (nsCSF) leaks (29% vs. 7%), while noting that the prevalence of OSA is likely underreported [5]. Due to these associations, it can be inferred that as the incidence of obesity and OSA continue to rise in the population, the incidence of sCSF leaks may also increase. Given that OSA has been shown to perpetuate changes that may result in sCSF, there may also be a role in the treatment of OSA in the prevention or management of sCSF leaks.

While the importance of diagnosis of OSA has been increasingly recognized as a comorbid condition in many systemic illnesses, there are a few studies that investigate the true prevalence of OSA in sCSF leaks. Polysomnogram (PSG) remains the gold standard for diagnosis of OSA but requires increased time and costs associated with the need to perform an in-person assessment at a sleep center. Prior studies that have investigated sCSF leaks have utilized PSGs to assess for OSA. Home sleep apnea testing (HSAT) presents many potential advantages compared to conventional PSG including greater accessibility, sooner treatment initiation, and cost reduction. Although HSAT has been shown to potentially underestimate OSA severity, they have demonstrated a high sensitivity to diagnosing mild or greater OSA when compared with PSG [6]. Furthermore, the use of home sleep apnea testing (HSAT) to screen for OSA in patients with sCSF leaks has not been reported.

In this paper, we review the current literature regarding OSA and sCSF leaks, describe our institutional experience, report objective sleep study findings, and demonstrate the reliability of HSAT to screen for OSA in patients with sCSF leaks.

## Materials and methods

Literature review

We conducted a review of the literature using Ovid, Cochrane library, and Pubmed for studies of spontaneous lateral skull base defects, which include meningoceles, sCSF otorrhea, and sCSF leaks, that also discussed the prevalence of BMI and OSA. Search terms included: “spontaneous”, “lateral skull”, “OSA”, “sleep apnea”, “encephalocele”, “CSF leak”, “otorrhea”, “temporal bone”, “mastoid”, “tympani tegmen”, “BMI”, “ICP”, “IIH”. Articles were excluded if they discussed non-spontaneous causes of CSF leaks (trauma, infection, malignancy, etc) or were not written in the English language. We compiled thirteen studies regarding OSA and sCSF leaks [4-16] and compiled data on a number of patients, age, sex, BMI, obesity (BMI > 30 kg/m^2^ ), and OSA prevalence into a standardized electronic data collection sheet. Weighted means for the compiled papers were determined by comparing proportional averages from each study with the total number of patients from all studies. The range of means was determined by reporting the maximum and minimum means from the 13 studies.

Sample selection

**Figure 1:**
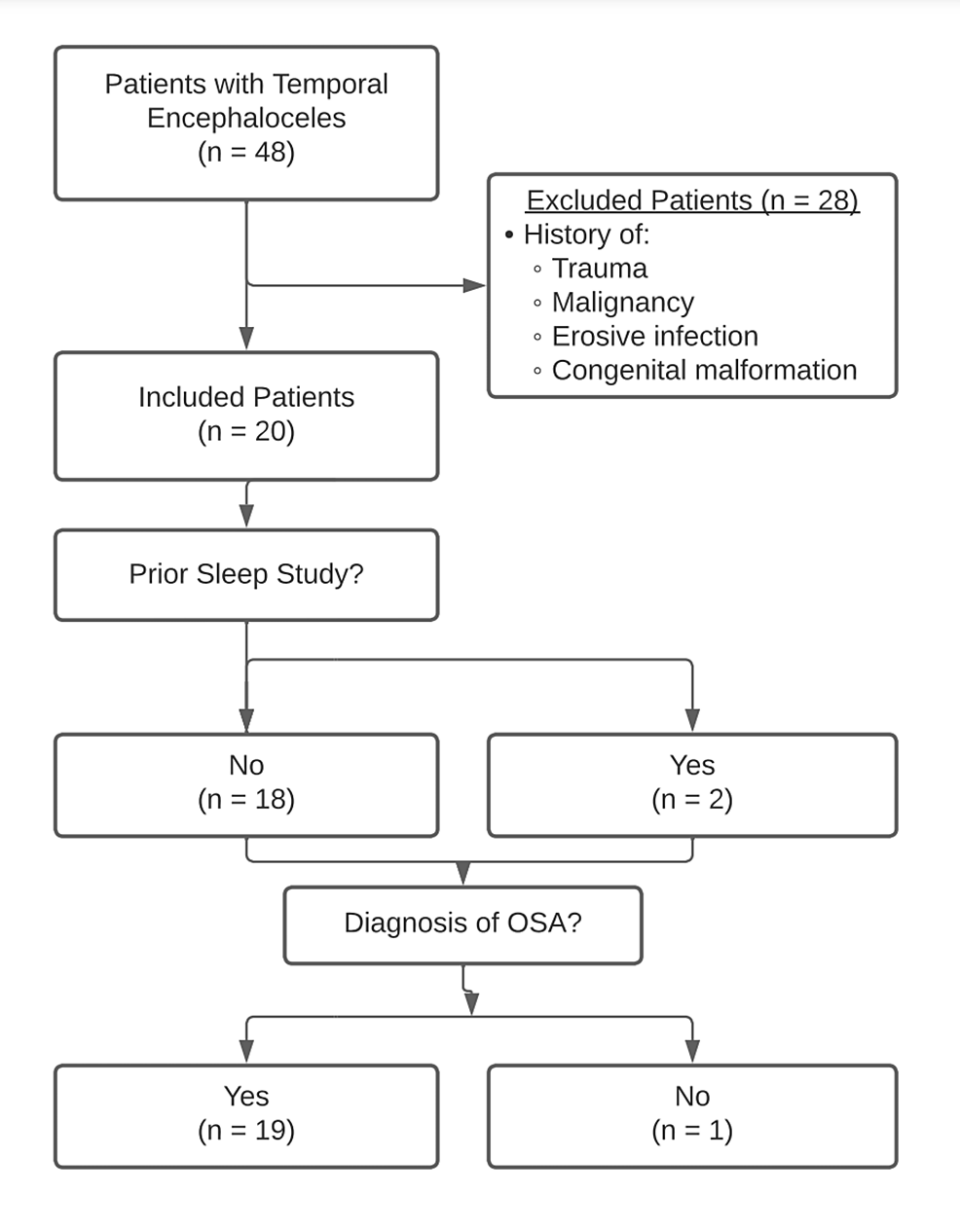
Flow Diagram of Patient Selection and Subsequent OSA Diagnosis OSA: Obstructive sleep apnea

Forty-eight patients with temporal bone encephaloceles requiring lateral skull base repair for CSF leak between 2013 and 2020 from a single academic center were identified. Twenty-eight patients were excluded for the determination of non-spontaneous leaks based on the reported history of trauma, malignancy, erosive infection, or congenital malformation. Following exclusion, 20 patients were included in the initial analysis (Figure 1).

To provide consistency when comparing data from conventional PSG and HSAT, the “apneic events index” was used to describe the apnea-hypopnea index (AHI) for PSG and the respiratory event index (REI) for HSAT results.

Statistics

Data were compiled into GraphPad Prism version 9.0 (GraphPad Software, San Diego, CA) and percentages and means were calculated. The results were shown as descriptive statistics without further statistical analysis. Graphs were generated for a visual demonstration of the data. MedStar Health Research Institute (MHRI) Institutional Review Board (IRB) approved the study with IRB# STUDY00000497.

## Results

Our literature search revealed key demographics for patients with spontaneous lateral skull base defects (Table 1).

**Table 1:** Literature Review on OSA and sCSF Leaks OSA: Obstructive sleep apnea; sCSF: spontaneous cerebrospinal fluid

Study (Year)	Number of Patients	Average Age (years)	Sex (% Female)	Average BMI (kg/m^2^)	Obesity (% Obese)	OSA Prevalence (%)
Illing et al. (2014) [6]	59	50.0	81.0%	37.7	83.0%	
LeVay et al. (2008) [5]	14	56.8	21.4%	35.1	92.9%	28.6%
Brown et al. (2004) [7]	9	65.9	66.7%			
Kenning et al. (2012) [16]	23	55.1	52.2%	33.2		
Kim et al. (2014) [8]	16	59.5	56.3%	40.7	100.0%	
Kutz et al. (2008) [9]	17	61.0	70.6%	36.0		
Melo et al. (2014) [10]	6	47.2	100.0%			
Nelson et al. (2016) [11]	60	57.5	68.3%	37.5		43.3%
Son et al. (2013) [12]	33			37.0		
Stevens et al. (2016) [13]	48	60.1	79.2%	35.7		
Stucken et al. (2012) [14]	11	61.6	72.7%	33.4	81.8%	
Vivas et al. (2014) [15]	32	56.0	68.8%	35.0	66.0%	
Rabbani et al. (2018) [4]	21	56.3	71.4%	35.3	81.0%	83.3%
Present study	20	57.1	65.0%	35.9	75.0%	95.0%
Number of Subjects	349	349	316	334	153	95
Weighted Mean	26.8	51.2	69.6%	36.4	81.8%	50.0%
Range of Means	(6-60)	(47.2-65.9)	(56.3-100.0%)	(33.2-40.7)	(66.0-100.0%)	(28.6-95%)

From 13 included studies, the weighted means and range of means were age: 51.2 years (n = 349, range {47.2-65.9 years}, sex: 66.8% female (n = 316, range {56.3-100.0% female}), average BMI: 36.5 kg/m^2^ (n= 334, range {33.2-40.7 kg/m^2^}), obesity: 81.8% (n = 153, range {66.0-100.0%}), and OSA prevalence: 50.0% (n = 95, range {28.6-83.3%}) (Table 2).

**Table 2:** Patient Demographics

Subject	Age (years)	BMI (kg/m^2^)	Sex (% Female)	Laterality
1	47	50	F	Bilateral
2	76	33.6	M	Right
3	56	30.63	M	Bilateral
4	42	27.2	M	Right
5	61	30.18	F	Right
6	63	47.5	M	Bilateral
7	58	51.3	F	Left
8	48	32	F	Right
9	38	39	F	Left
10	59	36.4	M	Bilateral
11	57	54	F	Right
12	49	38	F	Left
13	73	25.22	F	Left
14	63	38	F	Right
15	66	26.95	M	Right
16	55	25.8	F	Left
17	67	27.86	F	Right
18	65	38.85	M	Right
19	69	31	F	Left
20	30	35.09	F	Left
Mean	57.1	35.9	65.0%	
Range	(30-76)	(25.2-54)		
Maximum	76	54		

**Figure 2:**
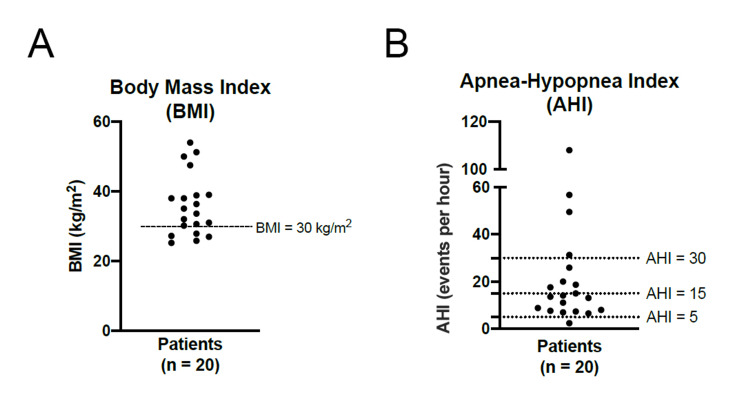
Scatter plots (2A) Scatter Plot of Patient BMI; (2B) Scatter Plot of Patient AHI; AHI: Apnea-hypopnea index

Twenty patients with sCSF leaks were included in this study following the exclusion of 28 patients for known trauma, malignancy, erosive infection, or congenital abnormalities. Of the 20 included patients, two patients had records of prior sleep study, and 18 received prospective sleep studies following diagnosis of sCSF leak. Table 2 describes the 20 patients with sleep studies: mean age 57.1 years (range 30-76 years), 65.0% female, mean BMI 35.9 kg/m^2^ (range 25.2-54.0) with 75.0% of patients clinically obese (BMI > 30 kg/m^2^) (Figure 2).

Of these 20 patients with sleep studies, there were 12 patients with HSAT (WatchPAT, Itamar Medical, Atlanta, GA, USA) and eight patients with overnight polysomnograms (PSG) in a sleep laboratory.

The two patients that had prior sleep studies were previously diagnosed with mild and severe OSA prior to the development of sCSF leak. Of the 18 that received prospective testing following diagnosis of sCSF leak, 94.4% (17/18) were subsequently diagnosed with OSA ranging from mild to severe. In total, 19 patients (95%) of the 20 patients were diagnosed with OSA ranging from mild to severe. The distribution of OSA severity was 10 mild OSA (52.6%), five moderate OSA (26.3%), and four severe OSA (21.1%) as determined by diagnostic criteria (Figure 2B.). Of the patients with study confirmed OSA, the results demonstrated mean apneic events 23.1 events/hour (n = 19, range 6.5-108), mean O2 saturation 93.3% (n = 15, range 88.0 - 97.0%), mean lowest O2 desaturation 85.1% (n = 18, range 64.0-92.0%), mean heart rate 69.6 beats per minute (bpm) (n = 17, 43.0-106.0 bpm) (Table 3). Of the twenty patients with sleep studies, the patients with HSAT (n=12) showed a lower mean number of apneic events compared to overnight PSG (n=8) (13.0 v. 35.8 events/hour).

**Table 3:** Sleep Study Data *Sleep study with OSA diagnosis prior to sCSF leak OSA: Obstructive sleep apnea; AHI: Apnea-hypopnea index; RDI: Respiratory disturbance Index; sCSF: spontaneous cerebrospinal fluid; PSG: Polysomnogram

Subject	OSA Diagnosis	Type of Sleep Study	Degree of OSA	AHI (events/hour)	RDI (events/hour)	Average O2sat (%)	Lowest O2sat (%)	Min Pulse (bpm)	Max Pulse (bpm)
1	Yes	Home	Mild	7		95	90	58	100
2*	Yes	PSG	Mild	8			90	54	79
3	Yes	PSG	Mild	13.1					
4	Yes	Home	Moderate	15		92	88		
5	Yes	Home	Moderate	25.9	26.9	94	78	52	122
6	Yes	PSG	Mild	11.1		90	84		
7	Yes	PSG	Severe	31.3	32.5	94	77	51	84
8	Yes	Home	Mild	6.5		97	92	46	94
9	Yes	Home	Mild	14.1	16.7		91		
10	Yes	PSG	Severe	108		88	74	25	99
11	Yes	Home	Moderate	20	21.1		87	44	108
12	Yes	Home	Moderate	17.6	18.8	93	89	49	61
13	Yes	Home	Moderate	18.7	19.4	94	87	36	69
14	Yes	PSG	Mild	8.8	22.4	94	88		
15*	Yes	PSG	Severe	49.5		92	82		
16	Yes	Home	Mild	13.6		93	64	51	115
17	Yes	Home	Mild	7.3	8.9	92	88	55	100
18	Yes	PSG	Severe	56.7	63.7	95	91	49	100
19	No	Home		2.4	4.3	95	92	47	81
20	Yes	Home	Mild	7.6	12.4	96	92	52	113
Mean	95.0%	60.0%		22.1	22.5	93.4%	85.5%	47.8	94.6
Range	(% OSA)	(% Home)		(2.4-108)	(4.3-63.7)	(88-97%)	(64-92%)	(25-58)	(61-122)

## Discussion

To emphasize the importance of recognizing obstructive sleep apnea (OSA) in the management of spontaneous cerebrospinal fluid (sCSF) leaks, we provide a brief review of obesity, sleep apnea, intracranial pressure, and associations with spontaneous skull base defects. Next, we discuss the current literature regarding the prevalence of OSA in patients with sCSF leaks. We then describe our institutional experience with 20 patients with sCSF and compare our data within the context of the current literature. Additionally, we highlight our experience utilizing home sleep apnea testing (HSAT) and discuss the reliability of this modality in screening for OSA in this population. Finally, we propose the importance of diagnosis and treatment of OSA in patients with sCSF leaks.

Overview and diagnosis of obstructive sleep apnea (OSA)

OSA is a disorder of recurrent episodes of nocturnal airway obstruction that causes decreased or absent airflow resulting in cortical arousal or oxygen desaturation. It is one of the most common sleep disorders, and it has been estimated that approximately 15-30% of males and 10-15% of females in the United States (US) and over 900 million individuals worldwide suffer from some degree of OSA [17]. Diagnostic criteria for mild or greater OSA include the apnea-hypopnea index (AHI) or respiratory event index (REI) > 5 episodes per hour of sleep. Traditionally, PSG was the gold standard for diagnosis of OSA, however, recent studies have demonstrated the reliability of HSAT to screen for OSA [6]. HSAT measures total recording time (TRT) compared to PSG which measures total sleep time (TST), precluding documentation of sleep stages and has been implicated in the potential under-reporting of OSA severity [18]. Despite this, HSAT has proven reliable in the diagnosis of OSA regardless of pretest probability [19]. Given the reduced costs, improved access to care and minimized risk of COVID-19 exposure, HSAT remains a viable option for the diagnosis of OSA.

Effects of obstructive sleep apnea on intracranial pressure and cerebrospinal fluid leaks

Elevated intracranial pressure (ICP) can be a devastating neurologic condition that has been shown to result in many sequelae, including cerebrospinal fluid (CSF) leaks [20]. It is estimated that longstanding ICP may be responsible for up to 45% of sCSF leaks [21]. One meta-analysis study reports that patients with CSF leaks are 4.73 times more likely to have OSA than control subjects (95% CI, 1.56-14.31; p = 0.006) [22]. Reports suggest that even mild OSA with minimal hypoventilation may significantly elevate cerebral blood flow and intracranial pressure [2]. Physiologically, the occurrence of apneas in patients diagnosed with OSA is thought to induce both transient and persistent elevations in ICP resulting in normal pressure hydrocephalus [23]. This process may result in remodeling of the skull base, favoring thinner areas of the skull base, resulting in bulging of the meninges forming encephaloceles and subsequent CSF leaks. Studies have shown that apneic episodes result in hypercapnia, hypoxia, and cerebral vasodilation, resulting in increased sympathetic activity, arterial pressures, total peripheral resistance, and cerebral blood flow and causes interference of CSF flow into the glymphatic circulation ultimately leading to increased ICP during apneic episodes and eventually awake ICP elevation [24].

During our review of the literature regarding the prevalence of OSA with spontaneous lateral skull base defects, we compiled 13 studies, of which three reported the number of patients with OSA. Rabbani et al. was the only paper that provided detailed sleep study data for patients with sCSF leaks, finding that 83.3% of patients with leaks had OSA [4]. LeVay et al. [5] and Nelson et al. [11] included previous histories of OSA (28.6% and 43.3%, respectively) [5]. In this combined analysis, it was determined that 50.0% of the included patients were diagnosed with OSA. Of the 13 studies, the weighted average of BMI was 36.4 kg/m with 81.8% of patients obese (BMI > 30 kg/m^2^). In comparison, in our experience with 20 patients who underwent sleep studies, the average BMI was 35.9 kg/m^2^ with 75.0% of patients being clinically obese (BMI > 30 kg/m^2^). Ninety-five percent (19/20) of patients with sleep studies had a diagnosis of mild OSA or greater. Of these patients with study confirmed OSA, the data demonstrated an average apneic event of 22.1 events/hour (n = 19).

Our findings show that the prevalence of obesity in all included patients (75.0% vs. 81.8%) is comparable to published studies with our study showing a greater proportion of patients that were diagnosed with OSA compared with the literature (95.0% v. 50.0%). Rabbani et al. (2018) is the only other study, to our knowledge, that reports detailed sleep study data with regard to sCSF leaks. In comparison, our study demonstrated similar patient demographics to Rabbani et al. with regards to gender (65.0% vs. 71.0% female), mean age (57.1 vs. 56.3 years), mean BMI (35.9 vs. 35.3 kg/m^2^), and prevalence of obesity (81.8% vs. 81.0%) (Table 4).

**Table 4:** Comparison of Demographic and Sleep Study Data of Current Study with Literature OSA: Obstructive sleep apnea; AHI: Apnea-hypopnea index

	Present study (n = 20)	Rabbani et al. (2018) (n = 21) [4]
Sex (% Female)	65.0%	71.4%
Mean Age (years)	57.1	56.3
Mean BMI (kg/m^2^)	35.9	35.3
Prevalence of Obesity (% Obese)	81.8%	81.0%
OSA Diagnosis (% OSA)	95.0%	83.3%
Type of Sleep Study (% Home)	60.0%	0.0%
Mean AHI (events/hour)	22.1	21.3
Mean Lowest O2sat (%)	85.5%	78.2%
Degree of OSA		
(% Mild OSA)	52.6%	33.3%
(% Moderate OSA)	26.3%	40.0%
(% Severe OSA)	21.1%	26.7%

Our study also depicts similar sleep study data including mean apneic event values (22.1 vs. 21.3 events/hour) and mean lowest O2 saturation (85.5% vs. 78.2% O2 saturation), however, our study reports higher prevalence of OSA (95.0% vs. 83.3%), higher proportion of HSAT (60.0% vs. 0.0%), and higher rates of mild OSA (52.6% v. 33.3%) compared to moderate (26.3% vs. 40.0%) and severe OSA (21.1% vs. 26.7%). When comparing HSAT (n=12) to PSG (n=8) in our cohort, there were comparable diagnostic rates (91.7% vs. 100%) although the mean apneic events were lower (13.0 vs. 35.8 events/hour). This supports that although HSAT may underreport the severity of OSA, it can still be used as an effective screening tool to potentially expedite the diagnosis and management of OSA in this population. Additionally, a combined 34.6% of the PSG (n=26) patients between the two studies were diagnosed with mild OSA, highlighting that even mild OSA may be associated with sCSF leaks and supporting the importance of making the diagnosis of OSA in this population.

This highlights that our results are compatible with the current literature and further demonstrates a high rate of OSA in patients with sCSF leaks, and is the first study to utilize HSAT to screen for OSA in patients with sCSF leaks. Although our study involves a limited number of patients, it supports the importance of OSA diagnosis and the use of HSAT as a screening tool when evaluating patients with sCSF leaks.

Limitations of the study

While our study attempts to describe and provide an institutional comparison with the current literature regarding OSA and sCSF leaks, there are many limitations to the study. First, our study includes relatively few subjects (n = 20), although is comparable to the only other known similar study (n=21). A larger study with additional patients would assist in elucidating our findings. As was previously mentioned, HSATs have been shown to underreport the severity of OSA and thus this may have resulted in underreporting of both the prevalence and severity of OSA among patients receiving HSATs. 

## Conclusions

Although the association between OSA and lateral skull base defects is incompletely understood, it has become apparent that OSA may lead to increased ICP and consequently lateral skull base defects. This study suggests an association of OSA in sCSF leaks and demonstrates the feasibility of HSAT. Further studies are necessary to better evaluate whether preemptively treating OSA reduces the occurrence of spontaneous skull base defects in susceptible patients or decreases the rates of recurrence following repair. Additionally, the role of treating OSA in the management of sCSF must be further studied, as this may provide an important consideration in the care of this patient population.
